# Suicide prevention in schizophrenia spectrum disorders and psychosis: a systematic review

**DOI:** 10.1186/2050-7283-1-6

**Published:** 2013-04-30

**Authors:** Tara Donker, Alison Calear, Janie Busby Grant, Bregje van Spijker, Katherine Fenton, Kanupriya Kalia Hehir, Pim Cuijpers, Helen Christensen

**Affiliations:** Black Dog Institute, University of New South Wales Hospital Road, Prince of Wales Hospital, Randwick, Sydney NSW 2031 Australia; Centre for Mental Health Research, Australian National University, Building 64, 63 Eggleston Road Canberra ACT, 2601 Australia; University of Canberra, University Drive Bruce, Canberra ACT, 2617 Australia; Department of Clinical Psychology, VU University, Van der Boechorststraat 1, 1081 BT Amsterdam, the Netherlands; EMGO Institute for Health and Care Research, VU University and VU University Medical Center Amsterdam, Van der Boechorststraat 1, 1081 BT Amsterdam, the Netherlands

**Keywords:** Suicidal behaviour, Suicide, Psychosocial treatments, Psychotic disorders, Prevention

## Abstract

**Background:**

The incidence of suicide is high among patients with schizophrenia spectrum disorders and psychosis. A systematic review was performed to investigate the effectiveness of psychosocial interventions in reducing suicidal behaviour among patients with schizophrenia spectrum disorders and psychosis.

**Methods:**

Cochrane, PubMed and PsycINFO databases were searched to January 2012. Additional materials were obtained from reference lists. Randomised Controlled Trials describing psychosocial interventions for psychotic disorders with attention placebo, treatment as usual (TAU), no intervention or waitlist control groups were included.

**Results:**

In total, 11,521 abstracts were identified. Of those, 10 papers describing 11 trials targeting psychosocial interventions for reducing suicidal behaviour in patients with schizophrenia spectrum disorders and psychosic symptoms or disorders met the inclusion criteria. Odds Ratios describing the likelihood of a reduction in suicidal behaviour or ideation ranged from 0.09 to 1.72 at post-test and 0.13 to 1.48 at follow-up.

**Conclusions:**

Psychosocial interventions may be effective in reducing suicidal behaviour in patients with schizophrenia spectrum disorders and psychosis, although the additional benefit of these interventions above that contributed by a control condition or treatment-as-usual is not clear.

## Background

Suicide risk is greatly increased in schizophrenia (Hawton et al. 2005) and is, in particular amongst males, the leading cause of premature death (Ösby et al. 2000; De Leo & Spathonis 2003). It is associated with personal and family tragedy. Suicide rates are estimated to be ten to twelve times higher than among the average population (Carlborg et al. 2010; Hassan-Ohayom et al. 2008), with as many as half of all patients with schizophrenia reporting a history of attempts (Breier et al. 1991; Harkavy-Friedman et al. 1999). Between 4 to 5% of patients complete suicide (Carlborg et al. 2010; Hor & Taylor 2010). Risk factors for suicide in this population group include previous attempts, being male, experiencing co-morbid Post Traumatic Stress Disorder (PTSD), and recent hospital discharge without treatment planning (Carlborg et al. 2010; Kasckow et al. 2011; Meltzer 2006; Tarrier & Picken 2011; Palmer et al. 2005). The risk of suicide in patients with schizophrenia is considered to be higher in the early course of the illness, especially within the first year (De Leo & Spathonis 2003; Kuo et al. 2005). Hawton et al. (2005) found that many of the important risk factors for suicide in schizophrenia were similar to those in the general population (e.g., mood disorder, recent loss, previous suicide attempts, drug misuse), but other risk-factors may be more specific to this population, such as fear of mental disintegration, agitation or restlessness, and poor adherence with treatment. Tiihonen et al. (2006) have confirmed, in a nationwide follow-up of individuals discharged from hospital after a first episode of schizophrenia, that not taking any regular antipsychotic medication was associated with a 12-fold increase in the relative risk of all-cause death and a worrying 37-fold increase in death by suicide (Hor & Taylor 2010).

Treatment for psychosis and management of the recovery of psychosis involves the use of anti-psychotic medication, Cognitive Behaviour Therapy (CBT), psychosocial treatments and combined methods. There is little evidence that antipsychotic medication has a suicidal preventive effect (Fakra & Azorin 2012), but for long-term treatment, clozapine, a second-generation antipsychotic, has been reported to reduce suicide attempts and completion rates in schizophrenia and schizoaffective disorders (Kasckow et al. 2011; Meltzer 2006). Clozapine is indicated for patients with schizophrenia whose psychosis is minimally responsive or intolerant to typical or atypical antipsychotic drugs at ordinary doses, or those who are at high risk of suicide because of its unique anti suicidal effect (Meltzer 2012). However, safety considerations (agranulocytosis, metabolic side effects and myocarditis) and the extra effort entailed in monitoring white blood cell counts to detect granulocytopenia or agranulocytosis limit the utilization of clozapine (Pompili et al. 2007).

A meta-analysis of CBT to reduce suicidal behaviour found a significant effect for CBT in reducing suicide behaviour in psychosis, but the effect was not significant if CBT was compared to another active treatment, indicating that the effect may be non-specific (Tarrier et al. 2008). Studies reporting the effectiveness of psychosocial treatments for reducing the risk of suicide attempts in psychotic patients show mixed results. Several studies reported no differences in suicidal behaviour (Barrowclough et al. 2010; Peters et al. 2010) while other studies reported decreased rates (Bateman et al. 2007; Tarrier et al. 2004). Suicidal behaviour in the early phases of psychosis has been reduced in an early intervention program (Bolton et al. 2007; Melle et al. 2006). Pharmacological interventions show that citalopram augmentation appears to reduce suicidal ideation in middle-aged and older participants with schizophrenia and subsyndromal depression (Zisook et al. 2010). However, pharmacological interventions require a minimum of six weeks to exert maximal efficacy (Fenton 2000), and for clozapine in particular several months may be required for the effects to become apparent (Kasckow et al. 2011). Therefore, an integrated approach of pharmacological and psychological interventions may be of particular importance, since evidence-based psychosocial interventions may decrease suicidal behaviour, decrease other risk factors of suicidal behaviour such as hopelessness (Power et al. 2003) and depressive symptoms (Peters et al. 2010; Turkington et al. 2002), and increase compliance to medication in patients with psychotic disorders (Bebbington & Kuipers 1994; Leucht & Heres 2006). However, non-systematic reviews describing the efficacy of combined treatment regimes in the prevention of suicide in patients with schizophrenia spectrum disorders or psychosis report inconclusive conclusions (Carlborg et al. 2010; Kasckow et al. 2011; Marshall & Rathbone 2011; Malmberg et al. 2001) and a direct suicide prevention effect is yet to be proven (Tarrier & Picken 2011).

Previous reviews (Carlborg et al. 2010; Hor & Taylor 2010) have not included all the available studies, or were qualitative. Moreover, literature on suicide outcomes is diverse and scattered, and suicide prevention interventions are sparse. The present study aims to conduct a systematic review of preventative psychosocial interventions for suicide in individuals with schizophrenia spectrum disorders and psychosis, as well as examining specific features of the psychosocial intervention that contributes to its effectiveness.

## Methods

### Definitions

A psychosocial intervention is defined as an intervention which provides psychoeducation, psychotherapy (including CBT or psychodynamic therapy), case management (including Assertive Community Treatment [ACT]), supportive counseling or community treatment. The intervention could be delivered in any setting, including secondary care settings, community centers, hospitals, and inpatient or day patient treatment units, and delivered through face-to-face, email, internet or post, and in an individual or group format. Studies were excluded if the intervention did not target patients with schizophrenia and schizophrenia spectrum disorders directly, but was aimed at mental health professionals or family members of affected individuals.

### Data sources and screening procedures

A database of 167 papers on suicide prevention was used, which was developed through a comprehensive literature search in which the Cochrane trial database, PsycINFO and PubMed databases were searched for articles published in the period 1800 to January 2012, with the key search terms ‘Suicid*’ OR ‘self-harm’ OR ‘self-poisoning’ AND ‘trial’ OR ‘intervention’ OR ‘prevention’. In addition, the search was limited for ‘humans’, ‘English’ and ‘peer-reviewed journals’. Separate searches for systematic reviews and meta-analyses were done for the PsycINFO and Pubmed database using similar key search terms. The identified titles and abstracts were screened for eligibility by two independent researchers. Full text copies of all potentially relevant papers, or papers where there was insufficient information in the abstract to determine eligibility, were obtained. Full text articles were further screened and discarded from further analyses if they met exclusion criteria. Reference lists of all previous systematic reviews and meta-analysis studies across all disorders were checked for potential papers. Data extraction of relevant papers was completed by two independent researchers, with disagreements resolved through discussion or with a third or in some cases fourth researcher. Authors of the included studies were contacted for additional data.

### Inclusion and exclusion criteria

Studies in which a psychosocial intervention targeted self-harm or self-poisoning, suicidal ideation, attempts, suicide, for participants with symptoms of a schizophrenia spectrum disorder or a diagnosis of a schizophrenia spectrum disorder were included. All studies were required to report mental health outcomes specific to self-harm (self-harm, self-poisoning) or suicide (suicidal ideation, suicide attempts). All were published in peer review journals. There was no restriction on the age of participants. Only trials with a randomized controlled design were included which incorporated a control condition (no intervention, waitlist; treatment-as-usual [TAU]) or in which a psychosocial treatment was compared to a pharmacological treatment. Interventions aimed with the primary purpose of collecting suicidal outcomes (e.g. just ratings of suicidal behaviour) were included as were those which included secondary outcomes of suicidal behaviour. Studies were excluded if it was not an intervention study or did not have a comparison or control group, if suicidal behaviour was not an outcome, or when interventions were purely pharmacological. Medication as a potential intervention was not included, largely because almost all individuals with psychosis are on maintenance medication. However, current medication was not used as an exclusion criterion.

### Study quality

Jadad`s quality criteria (Jadad et al. 1996) is a procedure to independently assess the methodological quality of a clinical trial. Based on these criteria, study quality was assessed against three key criteria: randomization; double blinding; and withdrawals and dropouts. Quality ratings range from 0 to 5, although intervention trials for mental health disorders rarely are rated above 3 as double-blind conditions are rarely achievable.

### Outcome measures

There are broad definitional issues around the nature of suicidal ideation and suicide behaviour. For this review we considered all suicide and related constructs as outcomes for review, and used the terms described by researchers of the individual articles. These terms were “self-harm attempts” or “self-poisoning”, “suicide ideation”, “suicide plans” or “suicide attempts”. Although self-harm and self-poisoning may not involve suicide intent, there is evidence that these behaviours may lead to suicidal behaviour (Joiner et al. 2009; Nock et al. 2010). The review also examined primary outcome variables including depression and psychotic symptoms. Primary outcome measures included reduction of self-harm or self-poisoning, suicidal ideation, plans, attempts scores as measured on suicidal ideation, suicide attempts or depression scales.

### Descriptive measures

A second aim was to identify factors which may have contributed to the success of the intervention. These include the psychological or other content of the intervention, the severity of the psychotic disorder, the method of delivery of the intervention, the length of intervention, the recruitment method and the nature of the intervention setting.

### Statistical analyses

When Odds Ratios (OR) were not reported in the study and data were available and extractable, between group effect sizes (Cohen’s *d* or Hedges’ *g*) for intervention and control groups were calculated by taking the difference between the mean post-test scores and dividing by the pooled standard deviation (Cohen’s *d*). The formula for Hedges g is similar but it accounts for an imbalance between the sample sizes of the two independent groups. The procedures of the Comprehensive Meta-Analysis software (CMA; version 2.2.021; Biostat Inc., USA) were then used to convert the effect sizes to OR. Analyses using CMA software showed a high level of heterogeneity of study populations and methodology. Due to the small number of studies and the big differences in patient characteristics, interventions and outcomes, we decided that pooling of studies was not possible. Hence, a formal meta-analysis was not conducted.

Authors were contacted to provide additional data if needed. Two papers (Bateman et al. 2007; Cunningham Owens et al. 2001) did not provide sufficient data to calculate OR or effect sizes.

## Results

### Search results

**Figure 1:**
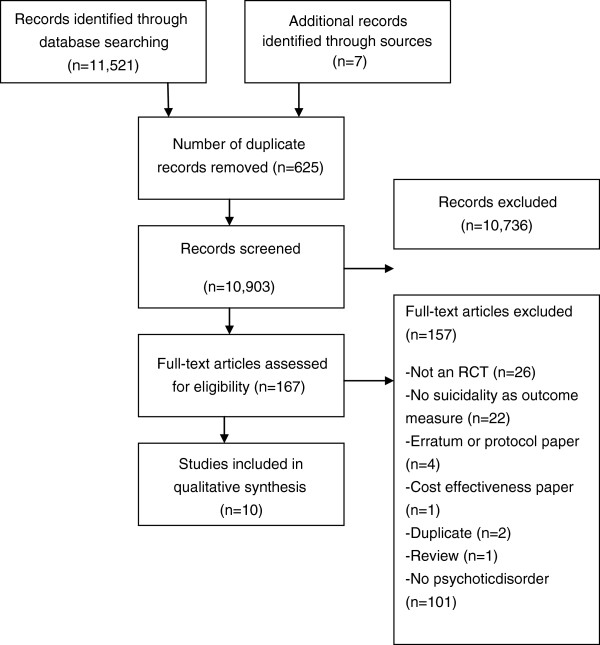
**Flow diagram for psychosocial interventions for suicidal behaviour in patients with schizophrenia spectrum disorders and psychosis.**

A total of 11,521 abstracts were examined (10,903 after removal of duplicates). Of these, 167 full text papers which were potentially eligible for inclusion were retrieved for further consideration, of which 157 were excluded. Ten trials (Barrowclough et al. 2010; Peters et al. 2010; Bateman et al. 2007; Power et al. 2003; Turkington et al. 2002; Cunningham Owens et al. 2001; Färdig et al. 2011; Grawe et al. 2006; Tarrier et al. 2006; Nordentoft et al. 2002) met the inclusion criteria. A further screening for possibly relevant references in systematic reviews or meta-analyses was conducted. Seven full text papers were retrieved for further assessment. However, none were included for final analysis as they failed to meet criteria (Figure 1).

### Characteristics of included studies

A total of 1,793 participants were recruited across all the studies. Of the 10 included studies, one study measured self-harm, and six measured suicidal ideation as a general outcome measure. Suicidal plans was used as an outcome in one study, attempts were assessed in three studies, and completed suicides in seven studies. Five of the 10 studies were CBT based (in addition to TAU). Of these, one study added integrated motivational interviewing to the CBT program, and one study, describing two trials used Supportive Counselling as content of the other intervention. One study delivered Cognitive Therapy, and one study delivered psychoeducation as the content of the intervention (aimed at improving understanding of the illness and acceptance of medication). Two studies used Integrated Treatment (IT) as content, and one study used the Illness Recovery Management (IMR) program. Nine studies incorporated TAU as a control group whereas one study used an attention-placebo. TAU in this population generally consists of anti-psychotic medication, outpatient and community follow-up, and access to community based rehabilitative activities. The setting of the studies was diverse. Eight studies recruited out-patients from secondary care or mental health clinics, whereas one study used in-patient or day-patients, and one study used in- and outpatients. Three studies used participants with a clinical disorder of schizophrenia, whereas five studies also included patients with other types of psychotic disorder, such as schizophreniform disorder, schizoaffective disorder, delusional disorder, psychosis NOS, schizoaffective disorder, acute or transient psychosis, induced psychosis or unspecific non-organic psychosis. One of these studies included co-morbid misuse or dependence of drugs/alcohol. One study included patients with ≥1 distressing and persistent positive symptom of psychosis and one study recruited patients with a first episode psychosis. One study was targeted at youth (15–25 years), whereas three studies included participants from age 16, and four studies used adults (18+ years). Two studies did not specify the age-range. All studies were delivered face-to-face, except for one study which used videos and booklets. All included studies used an individual format, except for one using a group format. Delivery length varied between one session and 24 months. All studies included participants with antipsychotic medication. However one study did not report this specifically. See Table 1 for an overview of the included studies.

**Table 1:** **Psychosocial studies on suicidal behaviour in patients with schizophrenic spectrum disorders and psychosis**

Trial	Content	Control	Population	Delivery type	Delivery format	Delivery length	Post-test/ follow up	Outcome measure	Outcomes (Intervention vs. control)	Effect size or O.R. (95% CI)	JQR
Barrowclough et al., 2010 (UK)	Integrated motivational interviewing and CBT plus TAU (n=164)	TAU (n=163)	Out-patients (>16 yrs) with schizophrenia, schizophreniform disorder or schizoaffective disorder and dependence on or misuse of drugs, alcohol or both	F2F	IND	12 mo	12/24 mo	Deliberate self-harm	Non-significant increase in self-harm in the intervention-group.	12 mo O.R.: 1.38 (0.65–2.96), *P*=.40; 24 mo O.R.: 1.48 (0.56–3.91), *P*=.43	3
Bateman et al., 2007 (UK)	CBT + MED (n=46)	Attention control + MED(n=44)	Out-patients (16–60 yrs) with chronic schizophrenia	F2F	IND	9 mo	9 mo/ 18 mo	Suicidal ideation (CPRS)	No suicides. Significant reductions in suicidal ideation at post-test and follow-up for CBT	n.a.	1
Cunningham Owens et al., 2001 (Scotland)	Educational intervention (n=61)	TAU (n=53)	Schizophrenic out-patients (16–64 yrs)	Video and booklets	IND	1 session	Follow up: 12 mo	Suicidal ideation (MADRS)	No suicides. Suicidal ideation increased (*P*<.001)	n.a.	2
Färdig et al., 2011 (Sweden)	IMR (n=21)	TAU (n=20)	Out-patient schizophrenia or schizoaffective disorder	F2F/ Power-point	Group	9 mo	9 mo/ 21 mo	Suicidal ideation (PECC)	Significant decrease in suicidal ideation at follow-up	PT: O.R.: 0.81 (0.28–2.33), *P*=0.69 FU: O.R.: 0.13 (0.04–0.41), *P<*.001	2
Grawe et al., 2006 (Norway)	IT (n=30)	TAU (n=20)	Out-patients (18–35 yrs) with schizophrenia, schizoaffective disorder or schizophreniform disorder	F2F	IND	24 mo	Post-test: 24 mo	Suicidal behaviour (attempts and suicide)	No suicides. Non-significant decrease on suicidal behaviour in intervention group.	O.R.: 0.95 (0.33–2.73), *P*=0.92	3
Nordentoft et al., 2002 (Denmark)	IT (n=156)	TAU (n=148)	In- and out-patients (18–65 yrs) with schizophrenia, schizotypical disorder, schizoaffective disorder, delusional disorder, acute or transient psychosis, induced psychosis or unspecific non-organic psychosis	F2F	IND	24 mo	Follow up: 12 mo	Tedium vitae, suicidal thoughts, -plans-, attempts (EPSIS II)	One suicide in the intervention group and one suicide or accident in the TAU group. No significant differences for suicidal behaviour	Thoughts: O.R.: 1.13 (0.54–2.35), *P*=.74. Plans: O.R.: 0.77 (0.30–1.98), *P*=.58. Attempts: O.R.: 0.95 (0.40–2.25), *P*=.91	2
Peters et al., 2010 (UK)	CBT (n=36)	TAU (n=38)	Out-patients (18–65 yrs) with ≥1 distressing and persistent positive symptom of psychosis	F2F	IND	6 mo	6 mo/9 mo	Suicidal Ideation (BSI)	No suicides. Significant reduction in being suicidal at 6 mo (but not at 9 mo)	6 mo O.R.:0.09 (0.02–0.53), *P*=.008; 9 mo O.R.:0.32 (0.07–1.6), *P*=.16	2
Power et al., 2003 (Australia)	Cognitive therapy plus TAU (n=31)	TAU (n=25)	Suicidal first episode psychosis out-patients (15–29 yrs)	F2F	IND	10 weeks	10 weeks/ 6 mo	Suicidal ideation (SIQ)	Two participants (one in each group) committed suicide. Significant greater average improvement on suicidal ideation in exp. group	PT O.R.: 0.29 (0.10–0.87), *P*=0.03	1
Tarrier et al., 2006 (UK)	CBT+TAU (n=101) and SC+TAU (n=106)	TAU (n=102)	In- or daypatients with schizophrenia, schizophreniform disorder or schizoaffective disorder, delusional disorder or psychosis NOS	F2F	IND	5 weeks	6 we/ 18 mo	Suicide and suicidal behaviour (self-harm, thoughts, attempts) (HoNOS)	Two suicides in the SC, one in CBT. Non- significant reduction in suicidal behaviour	6 we: CBT O.R.: 0.67 (.107–4.136) *P*=.66, SC: O.R.: 0.95 (.185–4.903), *P*=.9 18 mo: CBT: O.R.: 0.359 (.067–1.919) *P*=.23. SC: O.R.: 1.033 (0.301–3.55) *P*=.96	3
Turkington et al., 2002 (UK)	CBT + MED (n=257)	TAU + MED (n=165)	Out-patients (18–65 yrs) with schizophrenia	F2F + booklets	IND	2/3 mo	9 mo	Suicidal ideation (CPRS)	One suicide in TAU. Non-significant increase on the CPRS suicidal ideation score.	PT O.R.:1.72 (0.78–3.82), *P*=.20	2

BSI: Beck Suicidal Ideation Scale; CPRS: Comprehensive Psychopathological Rating Scale; EPSIS II: European Parasuicide Study Interview Schedule II; F2F: Face-to-Face; HoNOS: Health of Nation Outcome Scales; IMR: illness management and recovery; IND: individual; JQR: Jadad’s Quality Rating; MADRS: Montgomery Asberg Depression Rating Scale; MED: Medication; Mo: Months; N.A.: Not Applicable; NOS: Not Otherwise Specified; N.S.: Not Significant; O.R.: Odds Ratio; PECC: Psychosis Evaluation Tool for Common Use by Caregivers; SIQ: Suicide Ideation Questionnaire; SC: Supportive Counselling; TAU: Treatment As Usual.

### Methodological quality of included studies

Except for two studies (Bateman et al. 2007; Power et al. 2003), the quality of most studies, as measured by Jadad’s quality criteria (Jadad et al. 1996) was adequate (2–3 points). Every study met the first criteria of randomization, whereas half of the studies correctly described information of withdrawals or drop-out. None of the included studies was double-blind, a result which is commonly found in psychological intervention studies. Drop-out rates varied between 0% (Grawe et al. 2006) and 33% (Peters et al. 2010). Two studies did not report drop-out rate (Bateman et al. 2007; Power et al. 2003). Two studies (Bateman et al. 2007; Cunningham Owens et al. 2001) reported outcomes based on intention-to-treat analysis and could not be converted to Odds Ratios due to insufficient data reported in the studies. Eight studies reported completer analysis. Consequently, all Odds Ratios in this analysis are based upon the completer’s data.

### Effects of the interventions

#### Psychotic symptoms, depressive symptoms, hopelessness

All psychosocial interventions were associated with significant decreases in their primary outcome measures of psychotic symptoms (Peters et al. 2010; Bateman et al. 2007 described in Sensky et al. 2000; Grawe et al. 2006; Tarrier et al. 2006 described in (Lewis et al. 2002), insight into the illness (Turkington et al. 2002; Cunningham Owens et al. 2001), depression (Turkington et al. 2002), hopelessness (Power et al. 2003), substance use (Barrowclough et al. 2010) or overall symptomatology (e.g. mood disorders, anxiety disorders, somatoform disorders (Turkington et al. 2002)) compared to treatment as usual or over time (Peters et al. 2010; Power et al. 2003; Nordentoft et al. 2002).

### Self-harm

One study (Barrowclough et al. 2010) targeting self-harm found no significant differences (P>.05) in self-harm between the control group and intervention group for patients with schizophrenia, schizophreniform disorder or schizoaffective disorder and dependence on drug or alcohol, or alcohol misuse (post-test: OR: 1.38, 95% CI: 0.65–2.96, *P*=0.402; follow-up: OR: 1.48, 95% CI: 0.56–3.91, P=.433). Self-harm was measured with participant psychiatric case notes on admission to hospital for a reason related to psychosis or death from any cause.

### Suicidal ideation

Four of the nine studies that measured suicidal ideation (Peters et al. 2010; Bateman et al. 2007; Power et al. 2003; Färdig et al. 2011) found significant reductions (*P* < .05) on at least one measurement occasion in patients with psychotic disorders. Of the remaining studies, one (Cunningham Owens et al. 2001) reported significantly increased suicidal ideation in the psychosocial intervention compared to the control group (23.9% vs. 5.6%) and four (Turkington et al. 2002; Grawe et al. 2006; Tarrier et al. 2006; Nordentoft et al. 2002) found no significant difference (*P>*.05) in suicidal ideation between the intervention and treatment as usual groups. In two studies, a non-significant increase was found in suicidal thoughts (Nordentoft et al. 2002) and suicidal ideation (Turkington et al. 2002). In seven studies, suicide outcome measures were dichotomized (e.g.: 0=absence of suicidal ideation 1=presence of suicidal ideation; 0=no suicidal ideation 1=mild to severe suicidal ideation; 0=not present 1=at least once present). Overall, across these seven studies, Odds Ratios ranged from 0.09 to 1.72 at post-test and 0.13 to 1.48 at follow-up.

### Suicidal attempts

The three studies (Grawe et al. 2006; Tarrier et al. 2006; Nordentoft et al. 2002) examining suicidal attempts found no significant differences between the intervention group and treatment as usual on this measure.

### Completed suicide

There were no significant differences in completed suicides between psychosocial interventions and control groups in the eight studies which measured completed suicides as an outcome (Peters et al. 2010; Bateman et al. 2007; Power et al. 2003; Turkington et al. 2002; Cunningham Owens et al. 2001; Grawe et al. 2006; Tarrier et al. 2006;Nordentoft et al. 2002). Two suicides were observed in the studies of Nordentoft et al. (Nordentoft et al. 2002), Tarrier et al. (Tarrier et al. 2006) and Power et al. (Power et al. 2003), one in the study of Turkington et al. (Turkington et al. 2002).

## Discussion

All of the included studies in this review showed a significant overall improvement in primary outcome measures in psychosocial interventions for patients with schizophrenia spectrum disorders and acute psychosis over time and/or compared to treatment as usual. Furthermore, the reduction in suicidal behaviour for psychotic patients over time was present for the majority of the psychological interventions, but only 40% of these findings were statistically different to treatment as usual. This is in line with previous reviews on suicidal behaviour in general (Tarrier et al. 2008; Daigle et al. 2011) and studies with psychotic patients in particular (Carlborg et al. 2010; Hor & Taylor 2010; Kasckow et al. 2011). One explanation for the lack of difference may be the high quality of care in the TAU group. A contamination effect of other suicide prevention strategies introduced in the TAU groups may also be responsible for the effects. Another explanation could be that the individual studies had low power, given the low base rate of suicidal behaviour. In addition, the majority of the studies dichotomized the outcome measure in suicidal ideation, which may decrease the sensitivity of the measure and further increase the chance of a Type II error. Previous research examining psychosocial interventions specifically aimed at the prevention of suicidal behaviour has generally shown to be effective in reducing suicidal behaviour (Tarrier et al. 2008). In their meta-analysis, Tarrier et al. (2008) found that treatment is effective when directly focused on reducing some aspects of suicide behaviour but not when focused on other symptoms. However, in this review, the three studies reporting significant reductions in suicidal behaviour compared to controls, did not directly address suicidal behaviour in the treatment methods, whereas the two studies which did address suicide specifically showed significant reductions in suicidal behaviour over time, but not compared to TAU. Furthermore, given the finding that suicidal behaviour also decreased in TAU, it is unclear to what degree it is necessary to incorporate suicide specific modules into the treatment for patients with schizophrenia spectrum disorders and psychosis. Only one study (Cunningham Owens et al. 2001) found a significant increase in suicidal ideation. It provided an educational package for patients with schizophrenia and participants showed improved insight into the nature and consequences of the disease. Increased insight is not necessarily associated with suicidal ideation per se, as was shown in the study of Turkington et al. (2002) for brief CBT intervention in schizophrenia treatment. Therefore, an educational package alone might not be recommended, but should be accompanied with CBT or other coping skill strategies. Bearing in mind that measures of suicide behaviour are proxy measures for completed suicide (Tarrier et al. 2008), we were unable to draw inferences that psychosocial interventions can reduce actual suicide in patients with schizophrenia spectrum disorders or psychosis. More research is needed with large numbers of participants, to provide statistical power. The present study was unable to conduct a meta-analysis to combine data sets, due to sample heterogeneity. However, given that previous suicide attempts, depression and hopelessness are the largest risk factors for suicide in psychotic patients, reduction in these variables through psychosocial interventions are likely to prevent suicides. Despite significant reductions in suicidal behaviour, most of the study population samples remained at a high level of suicidal behaviour at the end of treatment compared to the general population, even after intensive and lengthy treatment.

Prediction and prevention of suicide in patients with schizophrenia spectrum disorders and psychosis is impaired by sample heterogeneity. The motivation for completing suicide may be very different for those in an acute psychotic phase compared to those in a recovery period. Likewise, suicidal processes may differ for those in early-onset compared to chronic patients. More studies with large sample sizes are needed to further our understanding of suicidal behaviour in psychotic patients and to improve treatments in suicide prevention.

Suicidal ideation was measured with different instruments (EPSIS, PECC, MADRS, CPRS, BSI, SIQ and HoNOS). Some studies used questionnaires in which one suicide specific question was used e.g. (Bateman et al. 2007; Turkington et al. 2002): CPRS), whereas others used subscales of suicidal behaviour or specific instruments or interviews for suicidal behaviour e.g. (Peters et al. 2010; Power et al. 2003; Nordentoft et al. 2002): EPSIS, SIQ, BSI) measuring thoughts, plans and/or attempts. These measures have demonstrated adequate internal reliability and concurrent validity e.g. (Beck et al. 1988; Reynolds 1988; Reynolds 1991; Orrell et al. 1999). Short questionnaires, such as the CPRS or HoNOS, require less time and expense (for administration and training) but limit the scope of suicidal ideation measured to obtain a broad range of data on suicidal behaviour, such as the EPSIS or SIQ. Few studies have investigated the psychometric properties of the suicide questionnaires among psychiatric and psychotic populations in particular (Orrell et al. 1999). Further studies using suicide assessment measures that target these populations are needed. In general, the heterogeneity of the suicide screening instruments hampers the generalization of findings, which may restrict knowledge of aetiology of suicide behaviour and treatment.

### Factors influencing the effectiveness of psychosocial interventions for suicidal behaviour in psychotic disorders

Given the small number of studies identified, it is difficult to isolate factors that influence the effectiveness of psychosocial interventions. However, we did note that the educational package and the two IT interventions (Cunningham Owens et al. 2001; Grawe et al. 2006; Nordentoft et al. 2002) failed to show significant differences compared to TAU, whereas three of the six C(B)T related interventions (Peters et al. 2010; Bateman et al. 2007; Power et al. 2003), and the one study using IMR (Färdig et al. 2011) did find significant differences, suggesting that content of intervention might influence effectiveness. In general, psychosocial interventions with a delivery length under 10 weeks did not show significant reductions in suicidal ideation (Cunningham Owens et al. 2001; Tarrier et al. 2006), whereas mixed results are found for delivery lengths of 10 weeks to 24 months (Barrowclough et al. 2010; Peters et al. 2010; Bateman et al. 2007; Power et al. 2003; Turkington et al. 2002; Färdig et al. 2011; Grawe et al. 2006; Nordentoft et al. 2002). Other factors such as type of delivery or format were not found to be strongly linked to outcome, a finding that suggests that the specific format and written delivery mode may not be critical. However, because of the paucity of the included studies, this observation would need to be tested further.

### Sustainability of results

Three studies showed a significant decrease in suicidal behaviour between intervention and TAU at follow-up (6, 18 and 21 months (Bateman et al. 2007; Power et al. 2003; Färdig et al. 2011)).

### Limitations

There are several limitations of this review that need to be addressed. First, because of the small number of eligible studies included in this review, in addition to the differences in samples, procedures and measures, the factors influencing effectiveness of an intervention were difficult to determine. Second, most studies included in this review measured suicidal behaviour as one outcome measure, whereas suicidal behaviour can be seen as comprising a range of outcomes, including thoughts, ideas, plans, attempts and death. This restricted any interpretations we could make about interventions for each type of outcome. The study was hampered by the range and quality of suicide outcome measures (Cuijpers et al. 2010). Third, we did not test for publication bias. However, given the significant differences in small sample size studies and non-significant results of larger sample size studies, we doubt that publication bias is likely to impact the conclusions drawn in our study. Fourth, the reported ORs in this systematic review were based on the completer’s data. Completer data is likely to yield higher OR as those retained in the study may be more likely than those who dropout to show positive effects. Therefore, results might be overestimated. Finally, only studies from peer-reviewed, English language Journals were included in this review. The effect of language bias minimally impacts the conclusions of systematic reviews (Wright et al. 2007).

### Future research

The results from this review have highlighted several ambiguities that require further examination. Further research into identification of mediators and moderators is needed, especially given the heterogeneity of the papers examined. Mechanism of change research could investigate to which degree specific suicide prevention modules need to be incorporated to yield maximum effects. More research into the development of buffering factors could be a further focus.

## Conclusions

In sum, this systematic review indicates that psychosocial interventions may have the potential to be effective in reducing suicidal behaviour in patients with schizophrenia spectrum disorders and psychosis, but the additional benefit of these interventions compared to treatment-as-usual are not clear. More research is needed in larger, better designed studies to be able to perform a formal meta-analysis.

## Abbreviations

CBT: Cognitive Behaviour Therapy
PTSD: Post Traumatic Stress Disorder
ACT: Assertive Community Treatment
TAU: Treatment-As-Usual
OR: Odds Ratios
IT: Integrated Treatment
IMR: Illness Recovery Management.
